# DIETARY INTAKE AND NUTRITIONAL RISK AMONG OLDER AMERICANS

**DOI:** 10.1093/geroni/igz038.3414

**Published:** 2019-11-08

**Authors:** Yeon Jin Choi, Jennifer A Ailshire, Eileen Crimmins

**Affiliations:** 1 University of Southern California, Los Angeles, California, United States; 2 Davis School of Gerontology, University of Southern California, Los Angeles, California, United States

## Abstract

A suboptimal diet and nutritional deficiency are among the leading causes of chronic diseases (e.g., cardiovascular diseases, metabolic syndrome, cancer, and osteoporosis), morbidity, and mortality. The objective of this study is to assess dietary intake and nutritional risk among older Americans. The dietary intake of 15 food and nutrients that are closely associated with the risk of poor health was assessed based on the dietary guidelines and nutritional goals for older Americans using a nationally representative sample of older adults (N=7,737) in the Health and Retirement Study Health Care and Nutrition Survey. The average consumption of most food and nutrients was out of the optimal range. For example, older men and women consumed 1.32-1.35 cups of dairy products and 1.23-1.29 ounces of whole grains, which is less than half of the suggested amount. The average consumption of sodium, on the other hand, was over 12 times greater than suggested dietary recommendation for older men and about 10 times greater for older women. The nutritional risk index (range: 0–15) was created by summing the number of dietary risk factors (not meeting the dietary guidelines and nutritional goals), the index scores for older men and older women were 11.05 (SD=2.31) and 10.09 (SD=2.60) respectively, suggesting the high level of nutritional risk. A healthy diet should be encouraged to prevent chronic diseases and improve the health of older adults. Nutritional education may be an effective way to promote a healthy diet.

